# Infectio: a Generic Framework for Computational Simulation of Virus Transmission between Cells

**DOI:** 10.1128/mSphere.00078-15

**Published:** 2016-02-10

**Authors:** Artur Yakimovich, Yauhen Yakimovich, Michael Schmid, Jason Mercer, Ivo F. Sbalzarini, Urs F. Greber

**Affiliations:** a Institute of Molecular Life Sciences, University of Zurich, Zurich, Switzerland; b University of Zurich, Zurich, Switzerland; c Medical Research Council, Laboratory for Molecular Cell Biology, University College London, London, United Kingdom; d Faculty of Computer Science, Technische Universität Dresden, Dresden, Germany; e Center for Systems Biology, Dresden, Germany; f Max Planck Institute of Molecular Cell Biology and Genetics, Dresden, Germany; University of Michigan

**Keywords:** infection spread, numerical simulation, hybrid modeling, multiscale modeling, cellular automata, particle strength exchange, diffusion, convection, advection, fluorescence microscopy, cell population, phenotypic properties

## Abstract

Viruses spread between cells, tissues, and organisms by cell-free and cell-cell mechanisms, depending on the cell type, the nature of the virus, or the phase of the infection cycle. The mode of viral transmission has a large impact on disease development, the outcome of antiviral therapies or the efficacy of gene therapy protocols. The transmission mode of viruses can be addressed in tissue culture systems using live-cell imaging. Yet even in relatively simple cell cultures, the mechanisms of viral transmission are difficult to distinguish. Here we present a cross-platform software framework called “Infectio,” which is capable of simulating transmission phenotypes in tissue culture of virtually any virus. Infectio can estimate interdependent biological parameters, for example for vaccinia virus infection, and differentiate between cell-cell and cell-free virus spreading. Infectio assists in elucidating virus transmission mechanisms, a feature useful for designing strategies of perturbing or enhancing viral transmission. The complexity of the Infectio software is low compared to that of other software commonly used to quantitate features of cell biological images, which yields stable and relatively error-free output from Infectio. The software is open source (GPLv3 license), and operates on the major platforms (Windows, Mac, and Linux). The complete source code can be downloaded from http://infectio.github.io/index.html.

**IMPORTANCE** Infectio presents a generalized platform to analyze virus infection spread between cells. It allows the simulation of plaque phenotypes from image-based assays. Viral plaques are the result of virus spreading from primary infected cells to neighboring cells. This is a complex process and involves neighborhood effects at cell-cell contact sites or fluid dynamics in the extracellular medium. Infectio differentiates between two major modes of virus transmission between cells, allowing *in silico* testing of hypotheses about spreading mechanisms of any virus which can be grown in cell cultures, based on experimentally measured parameters, such as infection intensity or cell killing. The results of these tests can be compared with experimental data and allow interpretations with regard to biophysical mechanisms. Infectio also facilitates characterizations of the mode of action of therapeutic agents, such as oncolytic viruses or other infectious or cytotoxic agents.

## Abstract (precis)

Infectio presents a generalized platform to analyze virus infection spread between cells. It allows the simulation of plaque phenotypes from image-based assays. Viral plaques are the result of virus spreading from primary infected cells to neighboring cells. This is a complex process and involves neighborhood effects at cell-cell contact sites or fluid dynamics in the extracellular medium. Infectio differentiates between two major modes of virus transmission between cells, allowing *in silico* testing of hypotheses about spreading mechanisms of any virus which can be grown in cell cultures, based on experimentally measured parameters, such as infection intensity or cell killing. The results of these tests can be compared with experimental data and allow interpretations with regard to biophysical mechanisms. Infectio also facilitates characterizations of the mode of action of therapeutic agents, such as oncolytic viruses or other infectious or cytotoxic agents.

## INTRODUCTION

Viruses are ubiquitous and infect all forms of life by mechanisms which are often specific for a certain virus type. Therapeutic interventions can target the entire virus replication cycle to either inhibit virus disease or enhance viral efficacy in therapeutic settings, such as oncolytic virotherapy (1–6). An area of increasing interest is virus transmission and spreading between cells. In an organism, this is a highly complex process involving a cloud of genetically nonidentical agents and polymorphic viruses, which enter and exit host cells (7–10). In addition, infections in organisms are tuned by cell autonomous and nonautonomous chemical modulators and many different cell types, immunity processes, or mechanical factors, such as fluid flux and shear stress (1, 11–15).

Viruses spread between cells by cell-free and cell-cell contact-dependent mechanisms. This involves lytic or nonlytic egress, superinfection repulsion, syncytium formation, or combinations of such mechanisms (16–19). An example of lytic cell-free spreading is provided by nonenveloped human adenovirus (HAdV), which has an icosahedral capsid about 90 nm in diameter and a double-stranded-DNA (dsDNA) genome of 36 kb (20, 21). HAdV plaques can be formed by cell-free virus, which is subject to passive mass transfer by diffusion and/or convection. However, the velocity field of the flow that carries viruses has remained difficult to estimate.

An example for complex cell-cell transmission phenotypes is vaccinia virus (VACV), an enveloped brick-shaped particle 360 by 270 by 250 nm in size with a large double-stranded DNA of ~200 kbp (22, 23). It replicates in the cytoplasm and forms two types of infectious particle, single-membrane-containing intracellular mature virions (IMVs) and double-membrane-containing cell-associated extracellular enveloped virions (CEVs), which upon detachment from the plasma membrane are termed extracellular enveloped virions (EEVs) (24, 25). VACV transmission is enhanced by actin tails, which result from CEV-induced back-signaling to the cell (26–29). This results in superinfection repulsion and enhances virus spreading (19). The number of CEVs that detach from the host cell is strain dependent. For example, the International health department-J (IHD-J) strain of VACV is known to produce significantly more EEVs than the Western Reserve (WR) strain (30). This feature has been measured in plaque assays under liquid conditions, but contributions from cell-to-cell and cell-free components have remained unknown (31).

Virus infectivity and transmission are classically measured by plaque assays in cultured cells (32, 33). However, these assays do not distinguish between different modes of transmission, such as cell-free and cell-cell transmission. This limitation may be circumvented by developing computational models and reverse engineering of the known spreading mechanisms. Here we present Infectio, a generalized software framework for virus transmission modeling. Infectio provides a hybrid multiscale model to simulate spatial dynamics of virus infections. It is a hybrid of cellular automaton (CA) and particle strength exchange (PSE) with a fluid mechanics component and a host cell component. Infectio models cell-free and cell-to-cell transmission features. It is implemented in MATLAB and supplemented with MEX/C for increased performance.

## RESULTS

### Generalized modeling framework.

A generic virus spreading model should be able to simulate plaque formation by a large variety of viruses. It should simulate different modes of transmission, including cell-free and cell-to-cell routes. Our previously published model for cell-free virus diffusion was a hybrid of CA and partial differential equations discretized using the PSE method (21, 34). To simulate cell-to-cell spread, we implemented neighborhood rules in our probabilistic CA (see Materials and Methods). We modeled the cell-to-cell spread of VACV on a descriptive level using the speed of plaque growth as an input parameter. In this case, we largely abstracted from all mechanisms of infectivity on the microscopic level (referred to here as microinfectivity) and explored if other input parameters besides speed of plaque growth would have to be considered.

To implement mechanistic parameters in a black-box abstraction model, we built in an option to make the speed of virus cell-to-cell spread dependent on the production of cell-associated virus by an infected cell and on the microinfectivity of the neighboring cells. Such a detailed model can make predictions about a number of VACV mechanisms that are currently discussed (see reference 19). The predictions from the model may then be tested by further experimental assays to validate microinfectivity properties.

**FIG 1:**
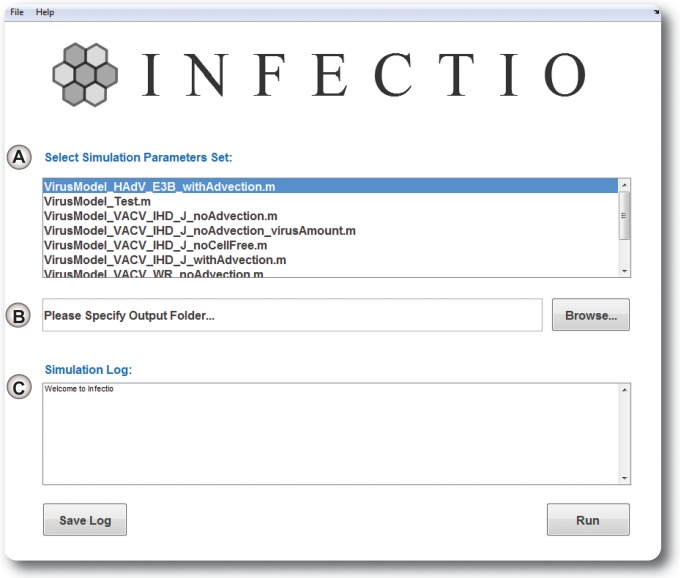
Graphical user interface for the Infectio software. (A) Selection of a predefined set of parameters for simulation. (B) Selection of an output folder. (C) Simulation log output.

Next, we enhanced the PSE component of our model by adding the possibility for particles to move along a defined velocity field. This feature allows simulation of not only homogeneous diffusion but also diffusion-advection of the cell-free virus in a defined direction. Finally, to enhance the usability of Infectio, we developed a graphical user interface (GUI) (Fig. 1). This interface allows one to choose specific simulation conditions, define the output path, or monitor the state of the simulation.

### Inference of convective bulk microflow properties from cell-free spreading patterns of adenovirus.

**FIG 2:**
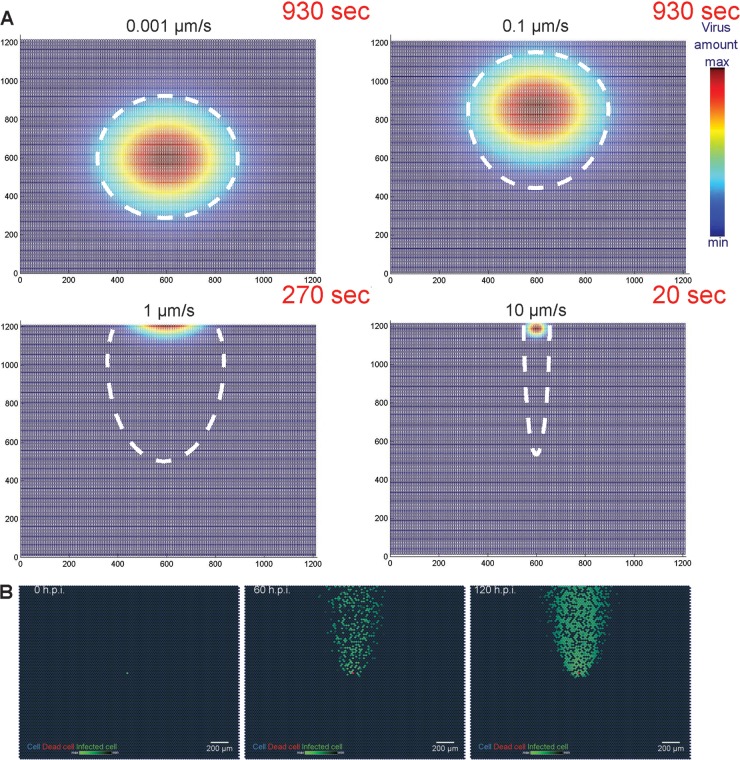
Addition of an advection term to the model simulates comet-shaped virus spreading patterns for adenovirus. (A) PSE particles with color-coded mass representing HAdV amounts reveal the concentration field behavior at different speeds of advection. Note that the simulation was stopped when the concentration reached the minimal infection threshold for HAdV; dashed lines show the shape estimate of the comets. (B) Still images from a simulated time-lapse infection with an advection term: cellular patterns of HAdV-infected cells closely resemble comets observed in microscopy. Here, advection speed was 0.5 µm/s.

To test the ability of Infectio to simulate phenotypes of both cell-free and cell-to-cell virus transmission mechanisms, we used published datasets of spatial dynamics of HAdV and VACV (21, 31). To infer the impact of convective bulk microflows, we performed spreading simulations of a replicating green fluorescent protein (GFP)-expressing adenovirus, HAdV-C2_dE3B_GFP, and for this we used different values of advection speed and left all other parameters invariant. We color-coded the infection intensity states of the cells and the virus amounts over time (Fig. 2; also, see Movies S1 and S2 in the supplemental material). The PSE simulations showed that advection velocities between 0.1 and 10 µm/s best reproduced the characteristic HAdV-C2_dE3B_GFP comet patterns (Fig. 2A; also, see Movie S2). Outside this range, the microflow was either too slow, yielding quasicircular plaques, or too fast, yielding very thin comets or low infection due to lack of virus binding to cells. An advection speed of 0.5 µm/s, however, yielded comets very similar to those observed in microscopy images for HAdV-C2_dE3B_GFP (Fig. 2B).

10.1128/mSphere.00078-15.3 Movie S1 Addition of an advection term to the model yields simulated comet-shaped virus spread patterns for HAdV. Plotted PSE particles with color-coded strength (virus amount) reveal the concentration field behavior at different speeds of advection. Still images are provided in Fig. 2. Download Movie S1, MOV file, 10.7 MB. Copyright © 2016 Yakimovich et al. 2016 Yakimovich et al. This content is distributed under the terms of the Creative Commons Attribution 4.0 International license.

10.1128/mSphere.00078-15.4 Movie S2 Simulated HAdV spreading time-lapse with an advection term. The cellular patterns of HAdV-infected cells closely resemble comets observed in microscopy; here, advection speed was 0.5 µm/s. Still images are provided in Fig. 2. Download Movie S2, MOV file, 3.2 MB. Copyright © 2016 Yakimovich et al. 2016 Yakimovich et al. This content is distributed under the terms of the Creative Commons Attribution 4.0 International license.

### Estimation of cell-free virus contribution to spreading of vaccinia virus.

**FIG 3:**
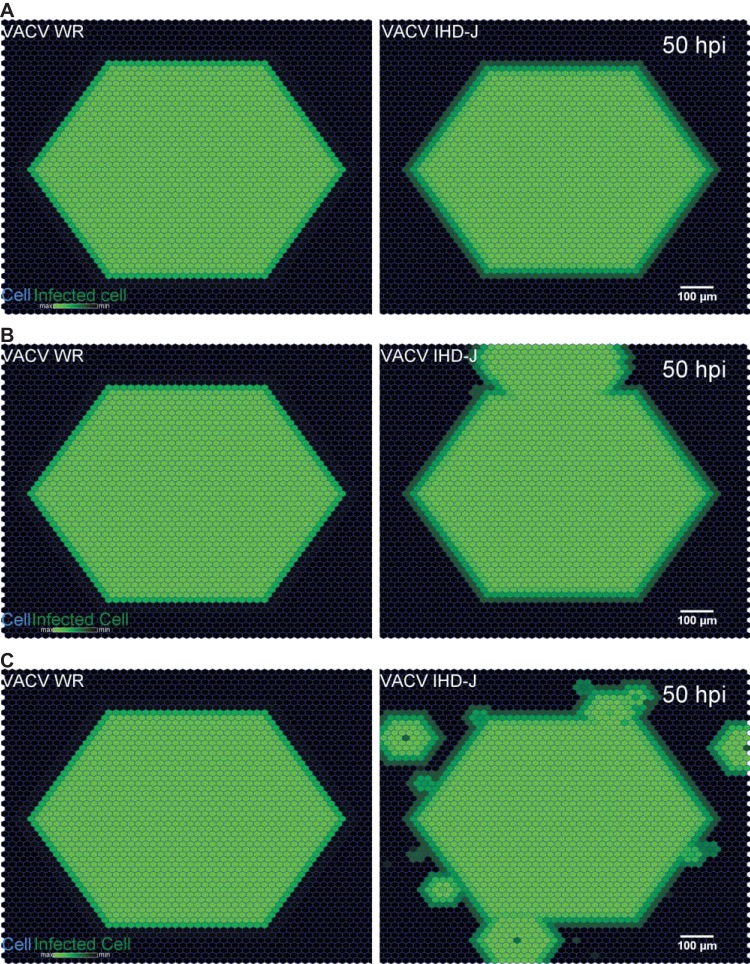
Vaccinia virus spreading patterns simulated under various conditions. (A) Cell-free spread of virus switched off. (B) Cell-free spread by diffusion-advection, where advection is directed north (along the OY axis). (C) Cell-free spread by diffusion only. Note that the hexagonal shape of the simulated plaque is due the geometry chosen for the shape of a unit cell. Circular plaques can be obtained with a more isotropic cell lattice (not shown).

To estimate how cell-free VACV contributes to the spread of infection, we have simulated a combination of cell-to-cell and cell-free virus transmission conditions of different VACV strains. The key parameters were either measured, estimated, or inferred from published datasets of WR and IHD-J strains, and graphical outputs were compared with phenotypes from microscopy experiments (31). We mimicked the experimental conditions in liquid or semisolid medium by introducing a directed advection flow of 0.5 µm/s and simulated VACV-WR or IHD-J spreading under three different conditions: condition A, without cell-free components; condition B, with diffusion and advection components; condition C, with diffusion but not advection flow (Fig. 3A to C).

Condition A mimicked the situation in semisolid medium under the assumption that VACV is too large to freely diffuse in the semisolid, and plaque formation occurs exclusively through cell-to-cell spreading (Fig. 3A; also, see Movie S3 in the supplemental material). As expected, both VACV-WR and IHD-J plaques were free of comet tails or satellite plaques. The diameter of the simulated plaque at 50 h postinfection (p.i.) was 1,092 µm for both VACV-WR and IHD-J, which is comparable to the diameters of experimental plaques, 1,098 ± 31 µm and 1,047 ± 27 µm, respectively. We conclude that although the simulation occurs in a simplified geometry, it reproduces the global spatial behavior of virus infections.

10.1128/mSphere.00078-15.5 Movie S3 Simulated time-lapse of VACV WR and VACV IHD-J spread with cell-free spread of virus switched off. Still images are provided in Fig. 3. Download Movie S3, MOV file, 4.4 MB. Copyright © 2016 Yakimovich et al. 2016 Yakimovich et al. This content is distributed under the terms of the Creative Commons Attribution 4.0 International license.

Condition B mimicked the situation where spread occurs in liquid medium, and cell-free virus is subject to advection-diffusion (Fig. 3B; also, see Movie S4 in the supplemental material). In the case of VACV-IHD-J, a number of satellite plaques appeared next to the initial plaque. These satellite plaques fused with the original plaque at 50 h p.i., and this gave rise to a comet tail phenotype. No satellite plaques were observed with VACV-WR at a scaling factor of 0.01. However, at a ratio (scaling factor) of 0.1, satellite plaques were observed at random locations around the original plaque (see Movie S5). We conclude that the amount of cell-free VACV-WR must be at least 100-fold lower than the amount of cell-associated virus to account for the absence of comet-shaped plaques.

10.1128/mSphere.00078-15.6 Movie S4 Simulated time-lapse of VACV WR and VACV IHD-J spread with cell-free spread through diffusion-advection and advection directed north, i.e., along the OY axis. Still images are provided in Fig. 3. Download Movie S4, MOV file, 4.5 MB. Copyright © 2016 Yakimovich et al. 2016 Yakimovich et al. This content is distributed under the terms of the Creative Commons Attribution 4.0 International license.

10.1128/mSphere.00078-15.7 Movie S5 Simulated time-lapse of VACV WR and VACV IHD-J spread with cell-free spread through diffusion-advection. Advection is directed north (along the OY axis), and the proportion of cell-free virus production for VACV WR was 0.1 instead of 0.01. Still images are provided in Fig. 3. Download Movie S5, MOV file, 4.5 MB. Copyright © 2016 Yakimovich et al. 2016 Yakimovich et al. This content is distributed under the terms of the Creative Commons Attribution 4.0 International license.

Condition C mimicked the situation in semisolid medium where cell-free VACV-IHD-J is subject to diffusion but not advection (Fig. 3C; also, see Movie S6 in the supplemental material). In contrast to the results obtained with VACV-WR, we observed a significant number of satellite plaques from the initial infected cell, consistent with the notion that VACV-IHD-J yields more cell-free virus than VACV-WR. If true, this would suggest that VACV-IHD-J freely diffuses through agarose and thereby gives rise to satellite plaques. However, this was never observed in wet-lab experiments (31). We conclude that the diffusion of cell-free virus released from a VACV-IHD-J plaques is restricted by 0.6% agarose gel. This shows that simulations by Infectio can predict the formation of viral plaques and help discriminate between different spreading mechanisms.

10.1128/mSphere.00078-15.8 Movie S6 Simulated time-lapse of VACV WR and VACV IHD-J spread with cell-free spread by diffusion. Still images are provided in Fig. 3. Download Movie S6, MOV file, 4.6 MB. Copyright © 2016 Yakimovich et al. 2016 Yakimovich et al. This content is distributed under the terms of the Creative Commons Attribution 4.0 International license.

### Availability and dissemination of Infectio software.

The large diversity of pathogens and the difficulty of growing certain viruses imply that a crowdsourcing computational effort could improve the models for virus transmission, for example, by using the most accurate experimental data and the most flexible software implementations. To enhance accessibility and utilize expertise from other researchers, we have deposited the Infectio software on the crowdsourcing platform GitHub (35). GitHub is based on the open-source version control system Git (GNU GPL V3 license), the current state-of-the-art system used by open-source software projects like Linux and Android. The source code of our modeling framework is available for download and for forking on GitHub under the GPLv3 open-source license, allowing freedom for researchers to work with the code. A community effort may help in building an ever-more-elaborate model of plaque formation for all known viruses. Placing different viruses in the same *in vitro* and *in silico* settings and comparing mutants, strains, and species in plaque formation and infection biology will enable novel insights for disease management and the use of viruses in therapies.

### Estimated software quality through software complexity.

The quality of software often negatively correlates with software complexity (36). For example, the number of defects that an *in silico* method contains correlates with the cyclomatic complexity, which is a measure of linearly independent paths through the source code for a program (37). Our analyses indicate that the complexity of Infectio is within an acceptable range, for example, lower than the complexity of the widely used CellProfiler software (Fig. S2). We maintained a high quality of the software by minimizing the complexity of our source code through iterative refactoring following addition of new features. To measure the complexity of the source code of Infectio, we used metrics including line count of commentaries (“Commentary”), line count of inline source documentation (“Help metrics”), and total line count (“Line count”) and combined this with cyclomatic complexity for each file of the source code. If the complexity increased upon the addition of new features, we performed refactoring of the source code to reduce it.

We compared the complexity of Infectio in three consecutive development versions to CellProfiler software (38) (see Fig. S2 in the supplemental material). For this purpose, the values of the metrics mentioned above were arranged in an array for each file of each version or software. For each metric, min-max normalization was applied to obtain values between 0 and 1 within the comparison set. This provided a complexity profile of the three consecutive versions of the Infectio software and CellProfiler software (see Fig. S2). For simplicity of the comparison, we computed an integrated measurement of the normalized metrics by averaging the values of all the metrics per version. The results showed that the initial version of Infectio (v0.1) had an average complexity of 0.0222. Upon refactoring, the complexity of v0.2 decreased to 0.0190. Upon introduction of additional features in v0.3, the complexity only slightly increased to 0.0207. For reference, the complexity of CellProfiler was 0.0268.

## DISCUSSION

Computational models have been used to identify infection contributions from viruses transmitted via cell-cell and cell-free mechanisms. This involved modeling of spatial dynamics of viral spread (39–42) and spatial behavior of the agents (43, 44). In particular, hybrid models involving CA, ordinary differential equations (ODE), or reaction-diffusion have been developed to simulate bacterial infections, conditionally replicating adenovirus in tumors or lytic virus replication in cell cultures (21, 45, 46). Our software Infectio implements a two-component spatiotemporal model and thereby reproduces the spreading phenotypes of three different viruses (HAdV, VACV-WR, and VACV-IHD-J). This represents a generic model of plaque formation applicable to any virus that has a known size and is able to form plaque phenotypes on cells grown in two-dimensional monolayers.

## MATERIALS AND METHODS

### Toward a hybrid model—cellular automaton.

Initially infected virus-spreading cells have received one plaque forming unit (PFU) and undergo a time- and PFU-dependent process of cell-free and cell-associated virus production according to the mathematical model with a ratio of cell-free to cell-associated virus (scaling factor) as discussed below (see equation 1; also, see Fig. S1 in the supplemental material). The amount of neighboring cells infected near an initially infected cell is determined by the plaque growth speed, measured in micrometers per hour. Each cell is represented by a hexagonal object, and in a monolayer, a hexagonal lattice represents biological cells, similar to the model described earlier (21). CA cells can be in a noninfected state (default), infected, dead, or lysed, where infected cells emerge from noninfected and dead cells or are lysed from infected cells. A single infected cell located at the center of the grid represents an initial infection, and adjacent noninfected cells may become infected by cell-free or cell-associated viruses (secondary infections). The probability of infection was measured experimentally and designated *P*_cell-free infection_, which attributes probabilistic cell infection values to the cellular automata. In this setting, we chose the cell-to-cell infection spread to be dependent on cell-associated virus and the microinfectivity of the neighboring cells.

10.1128/mSphere.00078-15.1 Figure S1  Fitting of VACV IHD-J extracellular virus production with a sigmoidal growth model. (A) Fitted curve. (B) Fitting residuals. (C) Fitting parameters and estimation of the goodness of fit. Download Figure S1, TIF file, 0.9 MB. Copyright © 2016 Yakimovich et al. 2016 Yakimovich et al. This content is distributed under the terms of the Creative Commons Attribution 4.0 International license.

10.1128/mSphere.00078-15.2 Figure S2  Complexity metrics arrays for Infectio compared to CellProfiler software. (A) An array of complexity metrics, including help metrics, cyclomatic complexity, counts of commentary lines and total lines computed for Infectio v0.1, v0.2, and v0.3. (B) An array of complexity metrics, including help metrics, cyclomatic complexity, commentaries lines count, and total line count, computed for widely used CellProfiler software. Here, all the values of the metrics were applied with min-max normalization of all values for the entire data set in a comparative manner. Download Figure S2, PDF file, 6.4 MB. Copyright © 2016 Yakimovich et al. 2016 Yakimovich et al. This content is distributed under the terms of the Creative Commons Attribution 4.0 International license.

### Particle strength exchange representing diffusive behavior of infectious agents in the extracellular medium.

Analyses of experimentally determined parameters of viral spreading in plaque assay indicated a contribution of cell-free adenovirus to infection (21, 31). It is, however, not known whether other viruses also freely diffuse through the agarose gel medium, as diffusion is dependent on virus size. We hence simulated free and limited diffusion conditions by coupling the advection-diffusion systems to our probabilistic CA representing the host cell population. The probability of cell-free infection is proportional to the amount of virus a cell receives from the PSE module. We introduced deterministic PSE methods mimicking diffusion-advection of extracellular virus (34). This is a continuum particle method discretizing the advection-diffusion PDE and is suitable for simulating the mass transfer of large numbers of virus particles typically present in the extracellular medium during cell-free spread. PSE was considered in a rectangular volume with an area equal to the dimensions of the CA cell, located above the cell and filled with equally spaced computational particles representing extracellular virus. The state of a CA cell is influenced by the amount of virus within the element above the cell, the movement of virus elements by advection under free-space boundary conditions allowing virus mass to exchange with neighboring PSE elements by diffusion. The method thus represents both bulk currents and local diffusion and accounts for the formation of comet-shaped and circular plaques, respectively (21).

### Algorithmic implementation of Infectio.

The algorithmic hybrid implementation of the cellular automaton and particle strength exchange (CA-PSE) methods is largely identical to the one published before (21). Here, we simulated the cellular monolayer using probabilistic CA (47, 48) on a hexagonal lattice. The latter is implemented in a spatiotemporal hybrid with PSE, a particle-based advection-diffusion numerical approximation method (34). PSE is based on replacing the Laplace operator with an integral operator that is subsequently discretized using particle locations as quadrature points (49). In the current implementation, an additional feature was implemented: the infection state is switched from “uninfected” to “infected” based on the parameter “speed of plaque growth,” allowing simulation of the cell-to-cell spreading mechansim. Further details can be obtained and discussed on the GitHub page under http://infectio.github.io or in the project help under http://infectio.github.io/help.html.

### Software implementation of Infectio.

The proposed method has been implemented as a software framework in the form of a stand-alone package. The main programming language was MATLAB with a well-structured modular design using MATLAB name spaces, which is reflected, for example, in the folder structures of the source code. Following this approach, it is easy to extend the framework by adding, setting files, or enumerating the model parameter space, each of which is different from a default scenario. Introduction of further model parameters or changes in the logics can be made using setting files. Additionally, to ensure user-friendliness of the software, we implemented a MATLAB GUID-based graphical user interface (GUI). Performance-critical parts were implemented as cross-platform C code, which is integrated using the MATLAB MEX technology. Application performance was further enhanced using parallel computing techniques through the shared memory model based on OpenMP 4.0 extensions. To ensure sufficient performance, the recommended hardware requirements include a high-end multicore workstation, or a multipurpose high-performance computer cluster.

The Infectio software is designed to take in a number of custom parameters defined by the end-user based on, e.g., experimental measurements as discussed below. An overview of input parameters, flags, and their default values are provided in Table 1. As a default output Infectio software provides frames of a time-lapse graphic representation of cells and final values of all the variables in the workspace saved as a .mat file. Additionally, one may save a graphical rendering of the PSE particles with color-coded particle strength (see the flags in Table 1). For experimental and troubleshooting purposes, one may save the state of any variable at any point of the simulation using “sensor” variables, which is deactivated by default but may be activated through a specific flag (see the flags in Table 1).

**TABLE 1:** Main input parameters of Infectio

Parameter	Type	Default	Description
cells_x	Integer	5	Model size defined by the number of the horizontal cells in the hexagonal lattice (*y* size is computed automatically to maintain the lattice)
pauseOnCAIterations	Flag (0 or 1)	0	Wait for key stroke or user input after every iteration
virus Flags.	Flag (0 or 1)	0	Should the images be displayed with or without PSE particles
plotImagesWithParts			
virusFlags.	Flag (0 or 1)	0	Advection: true, apply flow; false, do not
isAdvectionEnabled			
TotalTimeStepsHPI	Integer	10	Total time steps for the model to run in hours postinfection
virusFlags.	Flag (0 or 1)	1	Switches the cell-to-cell spread on and off
isSpreadCell2CellLimitedByTime			
virusFlags.	Flag (0 or 1)	0	Switches the cell-free spread on and off
isCellFreeSpreadEnabled			
initialC2cInfection	Integer	1	Number of initially infected cells
Sensor	Flag (0 or 1)	0	Save all workspace variable at certain hours p.i. during the simulation
CellDeathFlag	Flag (0 or 1)	0	Allow death of uninfected cells
CellLysDistFlag	String	“unif”	Cell lysis probability distribution flag for lytic cell-free spread: unif, norm, exp
SavePrticlesPlotFlag	Flag (0 or 1)	0	Saves the PSE particle plots
virusType	String	“VACV-WR”	Select one of the preset viruses; new presets for viruses with experimentally measured parameters can be definied in capse/src/matlab/+caps/+config/parameters.m and capse/src/matlab/+caps/+config/flags.m
PrimaryLysisFlag	Flag (0 or 1)	0	Defines whether the initially infected cell lyses to initiate spread

### Experimentally measured cell-free virus infection probability and average cell size.

To measure the probability of infection by cell-free VACV strains WR and IHD-J, we used the time-lapse titration data from a published data set (31). From these data, we estimated the fractions of infected cells depending on the amount of input virus at 12.3 h p.i., i.e., before the observable occurrence of the second round of infection. Both VACV-WR and IHD-J showed a dose-dependent increase of the fraction of infected cells. Fractions of infected cells were used as a look-up table, which allowed us to compute the probability of infection as a function of virus concentration using linear interpolation. We averaged the diameters of 40 BSC40 cells based on transmission light microscopy images (31) and found the average to be 22 ± 8 µm.

### Experimentally determined speed of cell-to-cell spread.

The speed of cell-to-cell spread was measured from the speed of radial growth (in micrometers per hour), detected by the emergence of the GFP signal around the initially infected cell. Although this measurement is probably less than the speed of virus transition between cells, we took it as an approximation of virus transmission from the originally infected cell to the neighboring cells.

### Experimentally determined cell-free and estimated cell-associated virus production.

VACV-WR produces 10 to 100 times less cell-free virus than IHD-J and 100 to 1000 times less cell-free than cell-associated virus (26, 31). Both strains produce comparable amounts of total infectious virus, and IHD-J produces about 10 times less cell-free virus than cell-associated virus. The amount of cell-free virus for VACV-WR is negligible within the time frame of our experiments, and hence we estimated the VACV-WR spreads between cells by cell-cell route only. To simulate the production of cell-free VACV-IHD-J progeny, we fitted the data from the supernatant titration experiment with a mathematical model of virus production. VACV growth curves have a sigmoidal shape, similar to the widely used one-step growth curves for other viruses (50). We hence fitted our experimental data with a sigmoidal model (see equation 1) using the least-squares fitting method. Since a negative concentration is nonphysiological, we constrained the parameter Bottom to be larger than or equal to 0. The fitting results and the residual plot are shown in Fig. S1A and B in the supplemental material. The best parameters obtained and the goodness of the fit are shown in Table 1.

(1) $$Y=\text{Bottom}+\frac{\left(\text{Top}-\text{Bottom}\right)}{1+10^{(\log{\;\text{EC}}_{50}\;-\;t)\;\times \;\text{Slope}}}$$

where *Y* is the virus amount, t is time after infection, and “Bottom,” “Top,” “EC_50_,” and “Slope” are the fitting parameters.

We compared the output of the model with infection spread of different viruses and obtained closely matching results. Next, we estimated the production rate of cell-associated viruses by conjecturing that VACV-IHD-J produces cell-free virus according to the model we fitted (see Fig. S1 in the supplemental material) using the best-fit parameters (see Fig. S1C). We also assumed that the production rate of other infectious forms follows the same model in either a scaled-up or scaled-down manner. The scaling factor for VACV IHD-J cell-associated virus production was 10. For VACV-WR, we assumed and used scaling factors of 0.1 and 0.01 (both conditions were tested) for cell-free virus and 10 for cell-associated virus. We assumed that both viruses produce the same amount of cell-associated virus (scale factor, 10).

### Estimated virus diffusion coefficient.

To estimate the diffusion coefficient we used the Einstein-Stokes relation (equation 2)

(2) $$D=\frac{k_{B}T}{6\pi \eta r}$$

where *k*_*B*_ is Boltzmann’s constant, *T* is temperature in kelvin, *η* is the dynamic viscosity of the medium, and *r* is the spherical radius of the diffusing particle.

As previously shown (21), the Reynolds number for our experimental setting is much less than 1, which justifies the use of the Einstein-Stokes relation under the conditions of our experiment. For the calculations with VACV, we took a spherical diameter of 270 nm, which corresponds to the longest dimension of a VACV particle. The computed diffusion constant for a spherical particle of this size is 1.218 µm^2^/s. Although VACV particles are barrel shaped and not spherical (23), we assumed in a first approximation that this difference in shape does not significantly influence the value of the diffusion coefficient.
