# Supplementary figures and images for: Infectio: a Generic Framework for Computational Simulation of Virus Transmission between Cells
Source: mSphere. 2016 Feb 10;1(1):e00078-15. doi: 10.1128/mSphere.00078-15 (PMC4863613; doi:10.1128/mSphere.00078-15)

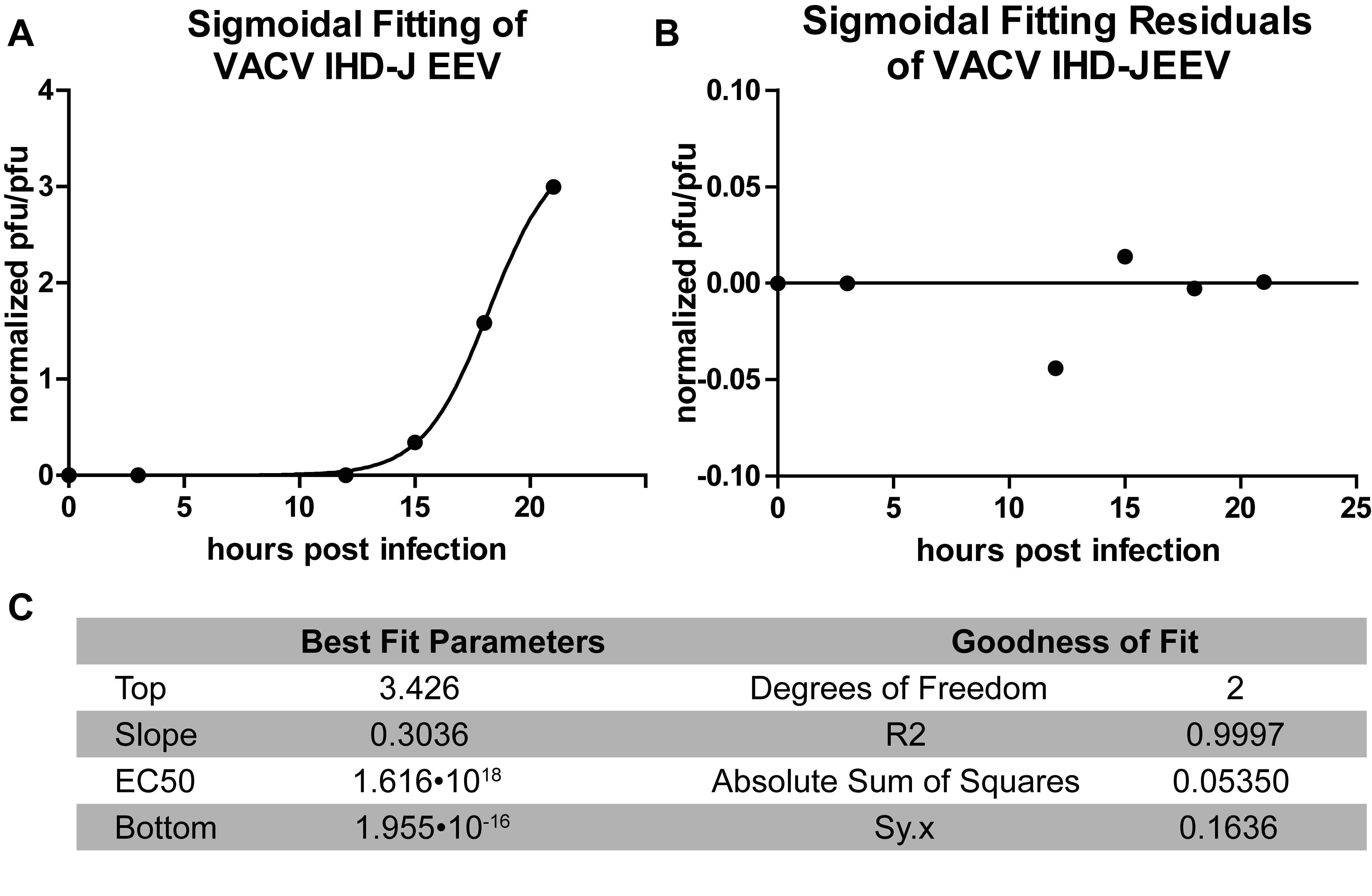

Supplement: Figure S1 [file sph001162007sf1.tif]

**Figure S2.** Complexity metrics arrays for Infectio compared to CellProfiler software

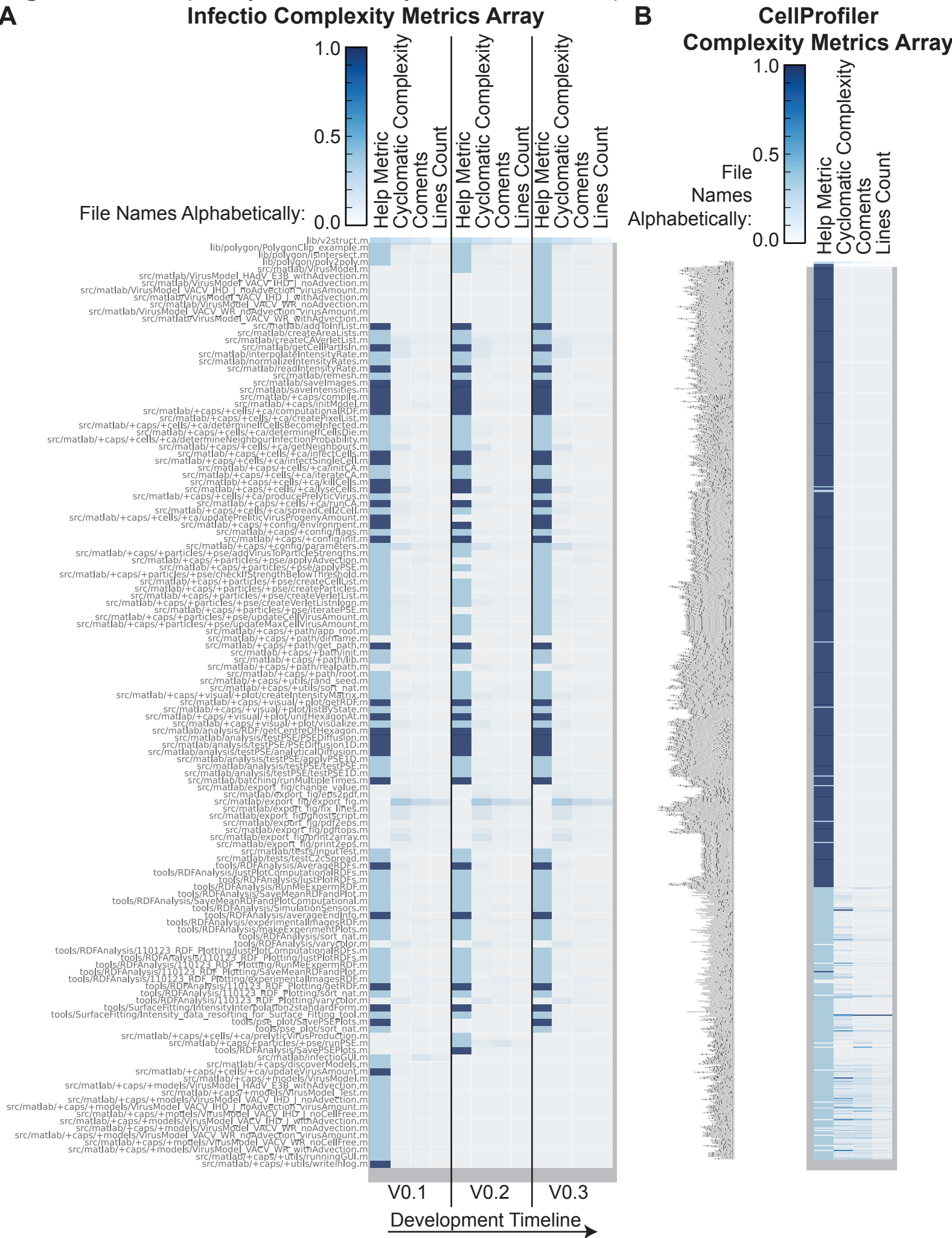

Supplement: Figure S2 [file sph001162007sf2.pdf]
